# A pilot study on the cutaneous effects of ethanol in a moisturizing cream on non-lesional skin of patients with atopic dermatitis

**DOI:** 10.1038/s41598-025-18487-9

**Published:** 2025-09-15

**Authors:** Murat Celikoglu, Christian Raab, Henning Vollert, Jürgen Harder, Xiaolin Liu, John F. Baines, Joachim W. Fluhr, Cornelia M. Keck, Victor H. P. Infante, Martina C. Meinke

**Affiliations:** 1 Department of Dermatology, Venereology and Allergology, Charité – Universitätsmedizin Berlin, Corporate Member of Freie Universität Berlin and Humboldt-Universität zu Berlin, Charitéplatz 1, Berlin, Germany; 2 Department of Pharmaceutics and Biopharmaceutics, Philipps-Universität Marburg, Marburg, Germany; 3 Bioactive Food GmbH, 23795 Bad Segeberg, Germany; 4 Department of Dermatology, Kiel University, Kiel, Germany; 5 Max Planck Institute for Evolutionary Biology, August-Thienemann-Str. 2, 24306 Plön, Germany; 6 Section of Evolutionary Medicine, Institute for Experimental Medicine, Kiel University, Arnold-Heller-Str. 3, 24105 Kiel, Germany; 7 Institute of Allergy, Charité-Universitätsmedizin Berlin, Corporate Member of Freie Universität Berlin, Humboldt-Universität zu Berlin, Berlin Institute of Health, Charitéplatz 1, Berlin, Germany; 8 Fraunhofer Institute for Translational Medicine and Pharmacology ITMP, Allergology and Immunology, Berlin, Germany; 9 Department of Dermatology, Venereology and Allergology, Center of Experimental and Applied Cutaneous Physiology, Charité – Universitätsmedizin Berlin, corporate Member of Freie Universität Berlin and Humboldt-Universität zu Berlin, Charitéplatz 1, 10117 Berlin, Germany

**Keywords:** Preservatives, Sensitive skin, Skin microbiome, Atopic dermatitis, Skin manifestations, Drug development

## Abstract

Ethanol is widely used in cosmetic formulations as a solvent, preservative, and penetration enhancer, yet its effects on atopic skin remain controversial. This study explores the impact of ethanol in skin care products on skin physiology, microbiome composition and subjective perception. A two-part investigation was conducted: (I) ex vivo analysis using porcine skin models exposed to varying ethanol concentrations, and (II) a double blinded, placebo controlled, randomized clinical pilot study on 9 patients with Atopic Dermatitis (AD) comparing creams with and without 12% ethanol. The ex vivo study revealed that ethanol concentrations above 15% negatively affected epidermal barrier integrity, increasing stratum corneum (SC) permeability and transepidermal water loss (TEWL). In the clinical trial, 12% ethanol demonstrated no significant adverse effects on SC hydration, erythema, pH, or TEWL over 30 days. Microbiome analysis revealed a localized increase in Xanthomonas species associated with ethanol use, while no significant community-wide changes were observed. The implications of increased Xanthomonas abundance in response to the application of a 12% ethanol cream for atopic dermatitis remain unclear. Subjective evaluations reported similar perceptions for both formulations, with no notable exacerbations in non-lesional AD skin. These findings indicate that ethanol in concentrations ≤ 12% is safe for atopic skin.

**Supplementary Information:**

The online version contains supplementary material available at 10.1038/s41598-025-18487-9.

## Introduction

Ethanol, an alcohol also known as ethyl alcohol, can be an important component of cosmetics^1^. It can act as a solvent, sensorial modifier, penetration enhancer and sometimes as a preservative^2^. However, the reputation of alcohols and ethanol as an important representative of alcoholic preservatives are not always positive^3^. People with sensitive skin or atopic dermatitis (AD) often avoid alcohol-based preservatives, alcoholic extracts, as well as fragrances, which are known allergens^4,5^. However, the literature to date shows no clear results regarding the effect of ethanol or other alcoholic preservatives in skin care creams and cosmetics, especially on atopic and sensitive skin^5^. This is often confounded with the oral intake of ethanol^2,3^. In patients with atopic dermatitis, alcoholic beverages can trigger or enhance itching.

Currently, every second natural cosmetic product contains alcohol (ethanol), as one of the five leading ingredients^6^. It is known that ethanol has a slight penetration-enhancing effect even at a concentration of 12.5% due to the attachment to the biphasic lipid layers in the skin by forming hydrogen bonds^7^. With concentrations higher than 25%, ethanol changes the basic structure of lipids in the skin. Concentrations of over 58% cause the formation of pores in the lipid layer and enable significantly increased penetration of active ingredients, potentially also into the skin^8^. The utilization of ethanol as a solvent in higher concentrations to enhance penetration of medications confirms the generally good tolerability of ethanol on the skin^9,10^. Nonetheless, intolerance reactions and sensitization are possible, even at lower concentrations^11^. Furthermore, topical application of ethanol in cosmetics is generally safe for healthy skin only – local or systemic toxic effects may occur in damaged skin, especially in children^2^. The effects on atopic skin have not been fully elucidated yet.

Atopic dermatitis (AD) is often seen as a leading example of a persistent skin disorder with a multifaceted underlying biology^4,5^. This condition can cause noticeable irritation (redness), itching, and discomfort on the skin. Additionally, it is linked with other atopic disorders, such as food allergies, allergic rhinitis, and asthma^12,13^. AD can impact people of all ages and is the most common skin disease among children, affecting about 15–20% of infants and young children and 2–3% of the general population in western countries^5^.

The role of the microbiome in atopic dermatitis continues to be debated. There is now a consensus that the skin microbiome plays a major role in the expression of atopic dermatitis^14^. Skin microbiome has been linked to the modulation of barrier function in several studies^15–17^. An important factor in relation to the skin microbiome and AD is the presence of Staphylococcus aureus (S. aureus). S. aureus colonizes the skin and therefore plays a role in the development and maintenance of AD. The exact pathophysiology and role of S. aureus is still unclear, but its relevance for disease burden, prolonged inflammation, and superinfections is clear^18,19^. Due to the progressing research on skin microbiome, studies are also needed on previously known secondary substances such as preservatives and their influence and effects on atopic skin.

Since the guideline-based treatment of atopic dermatitis starts with basic therapy delivered by emollients, it is fundamental to understand the effects of preservatives for atopic skin^20,21^. The use of emollients is also recommended in the relapse-free interval^5,20^. This highlights the importance of understanding the potential safety profile of ethanol. With this knowledge, dermatologists can provide accurate guidance to patients with atopic dermatitis regarding its use in daily basic skin care^22^. We would like to study whether the use of alcoholic preservatives could have an advantage in the treatment of microbial imbalances in AD patients.

In this paper the effects of ethanol in different concentrations on a porcine ex vivo skin model with intact skin was investigated in thin sections of biopsies using fluorescence microscopy. In the subsequent clinical study, a base cream preserved with 12% ethanol was tested in a double blind, randomized, controlled, half-side comparison pilot study vs. an ethanol-free base cream in a sterile dispenser. The aim was to investigate the effects of ethanol in a base cream on skin physiological parameters, the skin microbiome and subjective skin sensation. Questionnaires on stress and eating behavior were also collected.

## Materials and methods

### Base creams

The base cream was produced by URSATEC GmbH (Tholey, Germany) and provided by Bioactive Food GmbH (Bad Segeberg, Germany). The composition is present in Table 1, the formulation with ethanol had the same composition, except the reduction in the water amount represented by the addition of 12% of ethanol. Ethanol is used at this concentration for preservation purposes, aiming to replace other preservatives. The ethanol-free cream was kept sterile using an airless sterile dispenser, the cream containing 12% ethanol was stored in the same type of dispenser^23^.

**Table 1:** Composition of the base cream without added ethanol.

Chemical name	INCI	Supplier	function	Amount [g]
Purified water	Aqua	Freshly prepared	solvent	72,45
Dermofeel PA-12	Sodium Phytate	Evonik operations GmbH	stabilizer	0,1
Glycerin 86,5%	Glycerin, Aqua	Freshly prepared from Glycerin 99,5%	humectant	5
Cosphaderm x34	Xantham Gum	Cosphatec GmbH	emulsifier	0,25
Dermofeel GSC SG	Glyceryl Stearate Citrate	Evonik operations GmbH	emulsifier	3
Dermofeel TOCO 7 non-GMO	Tocopherol; Helianthus Annuus Seed Oil	Evonik operations GmbH	antioxidant	0,2
Palmester 3595	Caprylic Triglyceride	KLK Emmerich GmbH	emollient	6
Sunflower Oil	Helianthus Annuus Seed Oil	Bröckelmann& Co GmbH	emollient	5
Eutanol G	Octydodecanol	BASF AG	emollient	6
Lanette O	Cetearyl alcohol	Caelo GmbH	emulsifier	2

The pH of both cream formulations was measured retrospectively one year after completion of the pilot study. The ethanol-containing cream exhibited a pH of 4.79 (± 0.07, SD), while the ethanol-free cream showed a pH of 4.49 (± 0.07, SD). These results suggest that the pH values were likely between 4.5 and 5 at the time of application. During the storage period, the creams were sealed and stored in a controlled environment, shielded from light, with a stable humidity ranging from 40 to 60% and a temperature maintained 22,5 °C on average.

### Ex vivo measurements on Porcine skin

Ex vivo epi fluorescence microscopic images of porcine skin pretreated with ethanol in aqueous were taken followed by digital data analysis. Undamaged skin was compared with skin pre-damaged by acetone (AD-like lesions). Ethanol concentrations of 15, 18, 20, 80 and 96% were tested. In addition to fluorescence microscopy, stratum corneum (SC) thickness, penetration and stratum corneum autofluorescence were quantified according to Raab et al.^24^.

#### Skin models

Porcine ears from freshly slaughtered pigs were used for models of healthy skin. These were cleaned of dirt and blood and then subjected to a visual inspection for injuries, traumas or skin changes.

To verify skin integrity, the skin was examined using biophysical measurements (Courage & Khazaka Electronic GmbH, Cologne, Germany). Only ears were used in which the parameters of the measurements were in the range of healthy skin (transepidermal water loss: 5–12 g/m²/h and skin hydration: 60–70 [a.u.])^25^. The biophysical parameters were measured after cleaning and 30 min of acclimatization at a constant temperature of 21 degrees Celsius and humidity of 80%.

#### Epifluorescence microscopy

Autofluorescence imaging of the stratum corneum was performed as described by Raab et al.^24,26^. According to Pelikh et al.^27^ the fluorescence microscopic images were taken using inverted epifluorescence microscopy. An Olympus CKX53 microscope (Olympus Deutschland GmbH, Germany) was used with an Olympus DP 22 color camera (Olympus Deutschland GmbH, Germany) and a 130 W U-HGLGPS fluorescence light source (Olympus Deutschland GmbH, Germany). The images were taken at 200x magnification with the Olympus cellSens Entry software (Olympus Deutschland GmbH, Germany) with an exposure time of 50 ms and a lamp intensity of 100%.

#### Digital data analysis

Using ImageJ software (version 1.54 F, National Institute of Health, Gaithersburg, MD, USA), several data were extracted from the epifluorescence microscopic images.

**Fig. 1:**
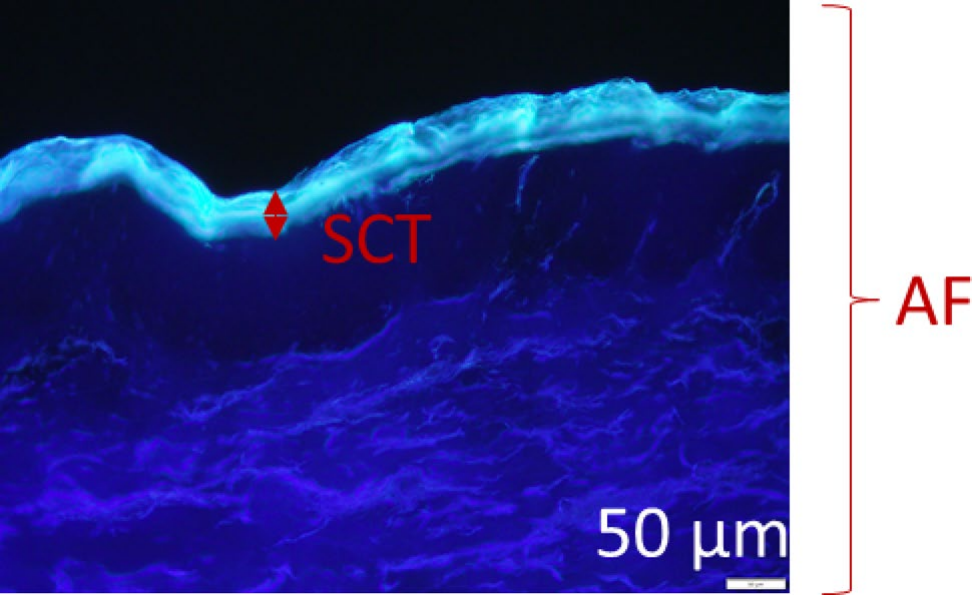
Illustration of SCT and AF in the original fluorescence image. The analysis was adapted from Pelikh et al.^27^.

From the original image (Fig. 1), the fluorescence of the entire image (AF) was determined as a measure of fluorescence intensity. With the length measurement vertical to the course of the SC, the thickness of the SC can be determined as stratum corneum thickness (SCT) in µm^27^.

### Patients and pilot study design

In total, nine participants with atopic skin were recruited for this study. The Ethical approval for the study was obtained from the ethics committee of the Charité – Universitätsmedizin Berlin (EA1/99/22) which were conducted according to the Declaration of Helsinki as revised in 2013. A written informed consent was obtained from all subjects. The trial were registered at the German Clinical Trial Register (DRKS) on the 24/06/2022 with the registration number DRKS00028916. All measurements were performed at the Department of Dermatology, Venereology und Allergology, Charité - Universitätsmedizin Berlin, CCP, during the period from December 2022 to March 2023. We utilized the SCORAD Index (SCORing Atopic Dermatitis) to evaluate the severity of AD. It is important to distinguish between diagnosing AD and assessing its severity^28,29^. The SCORAD score is used to quantify the severity of atopic eczema. Subjective points are awarded for erythema, edema/papule formation, oozing/crusting, excoriation, lichenification and dryness of the skin on a scale of 0–3. In addition, subjective symptoms such as itching and sleeplessness are rated on an analog scale of 0–10. The total extent of the eczema on the entire body surface is also considered. This score can have a maximum value of 103. Graduations are made between mild, moderate and severe eczema based on the score. A score below 25 corresponds to mild eczema, between 25 and 50 to moderate eczema while a score above 50 indicates severe eczema^29^.

Eight AD patients without acute eczema (SCORAD-Score of 0) and one patient with bilateral eczema (SCORAD-Score of 23,8 – mild eczema) in the antecubital fossae were included.

The average age of the patients included was 36,6 years (± 16,6 SD); 8 were female and 2 were male. Inclusion criteria were a confirmed medical history of atopic dermatitis (AD) without the need for systemic therapy, and recurrent eczema occurring 1 to 3 times per year. At the time of study enrollment, the skin had to be free of eczema for at least 30 days. In addition, no topical medications (such as glucocorticoids or calcineurin inhibitors) were allowed for at least 15 days before inclusion. Consequently, systemic therapy and recent topical treatment of eczema prior to study inclusion led to exclusion from the study.

The antecubital fossae on the left and right were examined and the cream was applied twice daily for 30 days for the study in a half-side comparison. The distribution of the two creams on the sides was randomized by two independent scientists, which were uninvolved in the present study, before. Recruitment and assessment of eligibility for inclusion were carried out by a clinician. On day 0 and 30 skin swabs were taken for microbiome analysis, a local SCORAD was recorded and skin physiological parameters were measured. An additional area of skin, the ventral side of the right upper arm, served as an internal control. This area was not treated or creamed in any way. All biophysical measurements and a control swab were taken at this site at day 0 and day 30. The same inclusion and exclusion criteria applied to the control site as to the antecubital fossae.

After the initial assessment, treatment with both creams could begin on the same evening. Before the final assessment, the last application of the creams had to be 24 h prior, and the skin was not to be washed or disinfected again.

We controlled the amount of cream applied per day using packages with dosage pumps. Each pump shot contained approximately 0.1 g cream. The cream was applied twice a day, morning and evening. Two pumps of each cream were used per application. Within the 30 days, the 9 patients used an average of 12.2 g (± 2,32 g) of the ethanol-containing cream formulation and 12.5 g (± 2,41 g) of the ethanol-free cream.

Patients were asked not to wash, disinfect or apply cream to the skin 24 h prior to the study visits.

Compliance and frequency of cream application were monitored using a checklist, which patients completed by ticking off each morning and evening after application of the study creams.

To evaluate the subjective perception of the two cream formulations, participants completed a questionnaire on day 30. The questionnaire included three questions, each rated on a numerical analog scale from 0 to 10, where 0 represented “very unpleasant” and 10 represented “very pleasant.” The questions focused on the cream’s scent, its sensory properties, such as ease of application, and the feeling of the skin after application.

The blinding was maintained until the completion of the statistical analysis. The identity of creams A and B was only revealed after the results had been received and analyzed, at which point it was disclosed which cream contained ethanol.

#### Stress and nutritional status

Atopic dermatitis is multifactorial, and flare-ups can be promoted by a wide variety of factors^13,14^. Important factors that are difficult to measure objectively include stress and the nutritional status. Regarding the nutritional status, we were particularly interested in the proportion of antioxidants in the diet in order to be able to assess the antioxidant level. By assessing the antioxidant and stress level during the study, we intended to identify possible triggers in addition to the cream application in cases of spontaneous exacerbation.

#### Skin physiological measurements

The SC hydration (capacitance), the erythema value, the pH value and the transepidermal water loss were measured. The Corneometer CM825, the Mexameter MX18, the skin pHmeter PM905 and the Tewameter TM300 were used for this purpose. The measurements were carried out in a room with a constant temperature of 22,5 degrees Celsius on average and a relative humidity of 40 to 60% after at least 15 min’ acclimatization. The devices were supplied by Courage & Khazaka Electronic GmbH (Cologne, Germany).

#### Microbiome analysis

Sk-3 S Isohelix^®^ Swabs were used to sample the skin microbiome. Before collection, swabs were soaked in a sterile, DNA-free buffer solution consisting of 50mM TRIS (pH 7.6), 1mM EDTA (pH 8.0) and 0.5% TWEEN-20. The skin swabs were taken in the antecubital fossae according to a predefined protocol. Twenty-five horizontal and vertical swab movements were performed in a field of 9 cm^2^ within the fossa for at least 30 s each. In addition, another swab was taken in the biceps area where no cream was applied within the 30 days of the study. The objective was to sample out the natural microbiome without the influence of the creams. Negative controls were performed by exposing the swabs to the ambient air for 10 s. In summary, a total of three swabs and a negative control were taken on day 0 and day 30 for each patient. The dry-stored (-80 °C) swabs were transferred to pathogen lysis tubes (Qiagen) under DNA-free conditions in a PCR workstation. After adding 650 µL ATL Working Solution (Qiagen), the tubes were incubated at 56 °C and 600 rpm for 10 min. Subsequently, the lysates were homogenized in a SpeedMill (Analytik Jena) for 1 min and centrifuged at 10.000 g for 2 min. 400 µL of the supernatant was transferred to a 2 mL of DNA-free Biosphere tube (Sarstedt) and the microbial DNA was analyzed using the QIAamp UCP Pathogen Mini Kit according to the manufacturer’s protocol and previously described^30^.

For quantitative analysis real-time PCR was performed with the v1-v2 variable region of 16 S rRNA primer set 27 F-338R adapted from Mesa et al.^31^ (27 F (5´-AGAGTTTGATCCTGGCTCAG-3′) and 338R (5´-TGCTGCCTCCCGTAGGAGT-3′). 16 S rRNA amplicon library preparation, sequencing, data processing and taxonomic classification was performed as previously described^32^. As additional control, microbial DNA from ZymoBIOMICS Microbial Community Standard (Zymo Research) was also isolated and sequenced^32^. Potential contaminant sequences were identified and removed using the decontam R package (frequency method, threshold = 0.1), retaining 3,196 different amplicon-sequence variants (ASVs). To address biases in relative abundance analysis for low-biomass samples, we applied quantitative microbiome profiling (QMP) using qPCR-based bacterial load estimates, transforming relative abundances into absolute abundances^33,34^. The skin bacterial community composition was then analyzed at the ASV level.

### Statistical analysis

The Shapiro-Wilk test for normal distribution was performed. Boxplots were visually inspected and a Z score was created and verified to see whether it exceeded the value +-3 in order to identify outliers. Both were carried out with SPSS (BM Corp. (2023). *IBM SPSS Statistics for Windows* (Version 30.0.0.0)).

Statistical outliers were not removed as long as they fell within an expected and plausible range where the values could reasonably occur and were not considered measurement errors. This applied to one measured value, which was plausible within the range of normal measurements which is why it was not removed (TEWL value 10,88 at day 30 after ethanol containing cream).

The significance level for the two-sided test was set at 0.05. To minimize an α-error, a multiple test correction was carried out using Bonferroni correction. In the case of normal distribution, a significance test was carried out using a paired t-test. All p-values below 0.05, after multiple testing correction, that remained within the significance level were considered significant. and denoted with an asterisk. (*). All values showed a normal distribution except for the TEWL. Non-normally distributed data were checked for significance using Wilcoxon tests.

The ex vivo data were statistically analyzed using a Kruskal-Wallis test followed by a Dunn post hoc test under the previously described conditions.

## Results

### Ex vivo investigation on porcine skin

On healthy porcine skin, the application of ethanol had concentration-dependent effects on the *SC*.

**Fig. 2:**
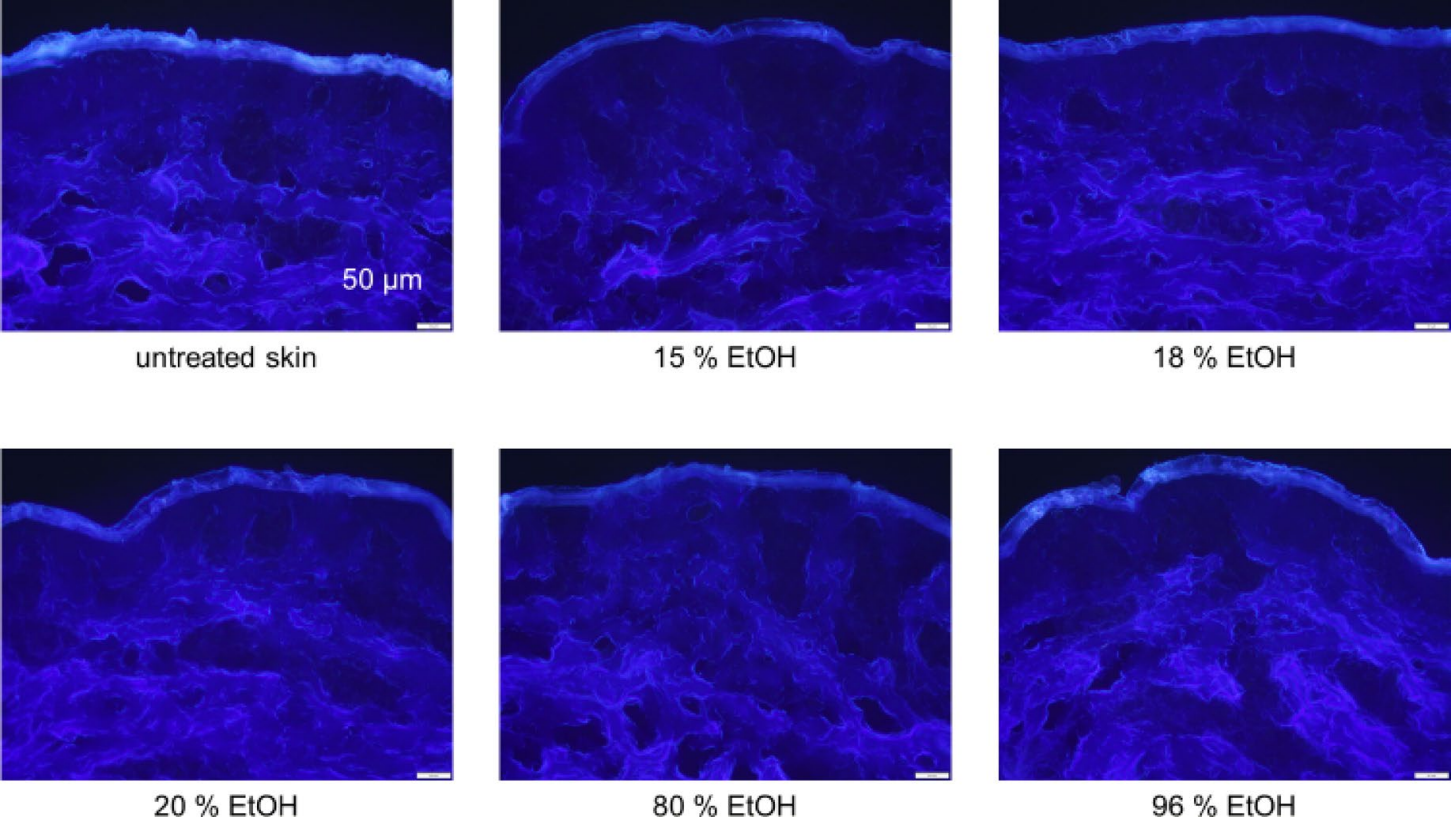
In-vitro fluorescence microscopic images of healthy porcine skin after application of ethanol concentrations of 15 to 96%. With increasing concentration, ethanol causes thinning of the SC and blurs the clear boundary between SC and epidermis, images captured at 200x magnification, scale bar = 50 μm.

**Fig. 3:**
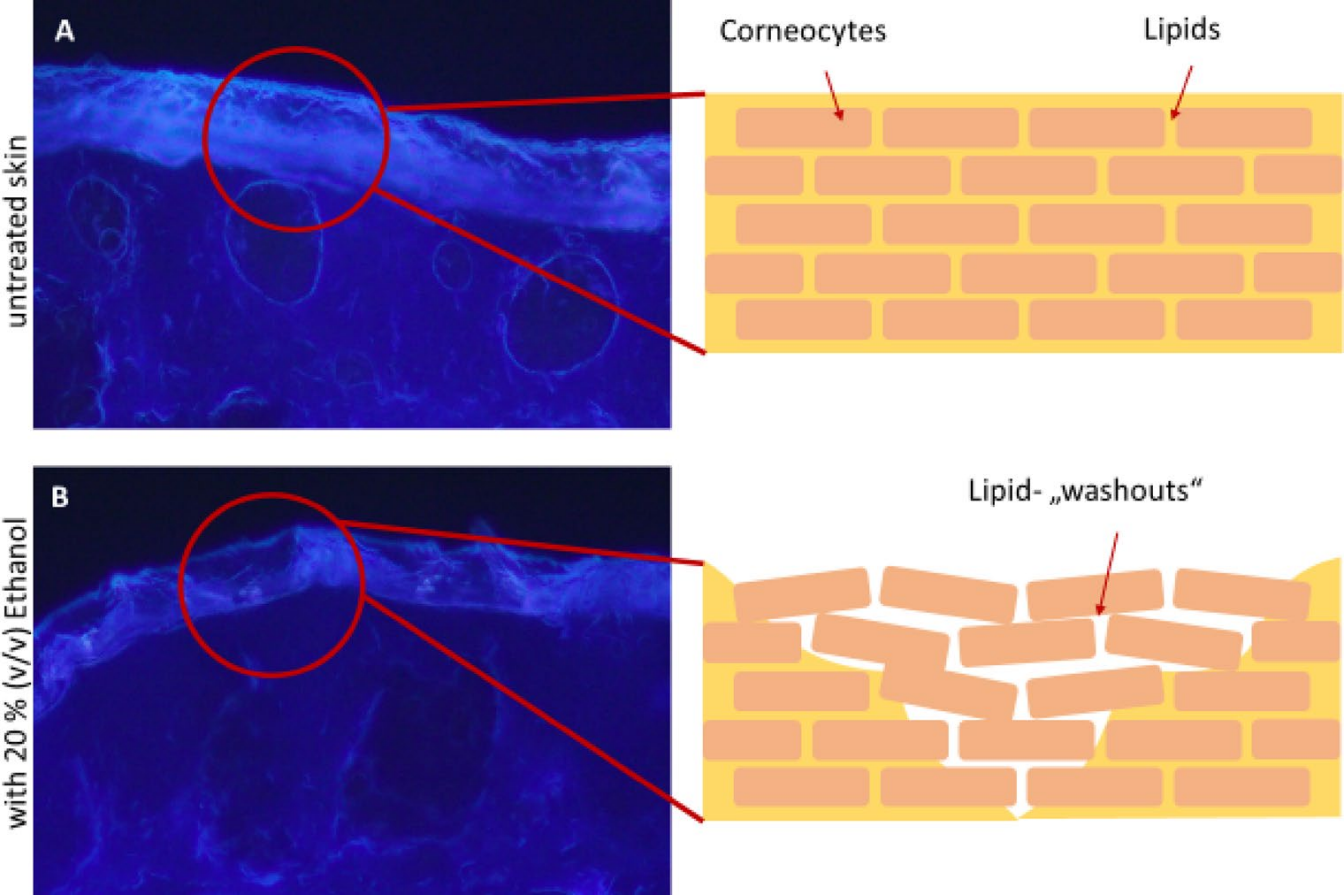
Fluorescence microscope images and schematic representation of the effects of ethanol on healthy skin. The brick-and-mortar model were used to illustrate the effects. Under the influence of ethanol, lipids are “washed out” between the corneocytes, causing the barrier to lose integrity. Scale bar = 50 μm.

As the ethanol concentration increases, the transition between the *SC* and the rest of the epidermis becomes irregular. Furthermore, stratum corneum discontinuities occur, which can be explained by the washout of SC lipids. (Figures 2 and 3). Quantification of the autofluorescence of the *SC* shows, there is generally a decrease in fluorescence intensity with increasing ethanol concentration, except at 80%. The germicidal property of the highly concentrated ethanol appears to increase the overall fluorescence (Fig S1).

**Fig. 4:**
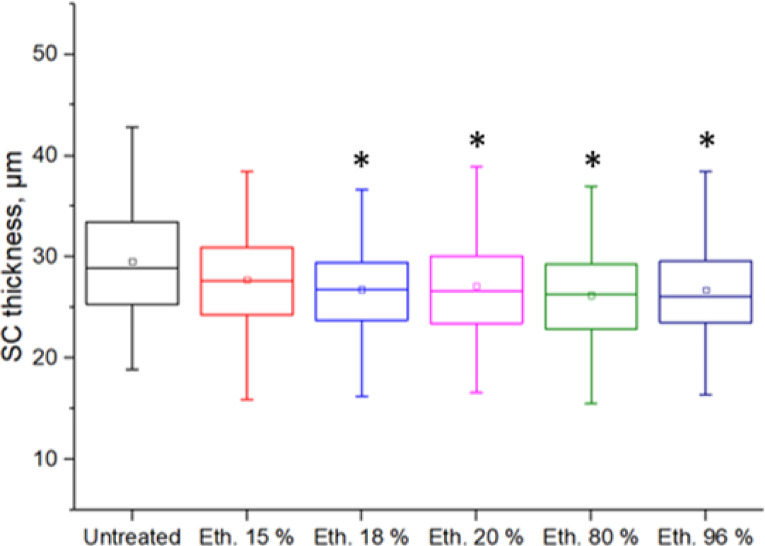
SC thickness after ethanol application with concentrations of 15 to 96%, *n* = 3 porcine ears with 280 different measured points per ear. The SC thickness decreased to different degrees with different ethanol concentrations. * *p* ≤ 0.05, tested against untreated.

At the same time, ethanol application thins out the *SC* of intact skin. For 15% ethanol, the thickness of the *SC* was reduced to 93% of the initial thickness (Fig. 4). This appears to increase water loss and the *SC* dries out. This can be explained by penetration of ethanol into the SC, epidermis and dermis and subsequently interfering with lamellar bilayers of the SC. However, a concentration of 15% showed only a very small reduction of the Stratum Corneum thickness (SCT) effect, which means that only minor penetration-enhancing effects can be expected (Figs. 2 and 4).

These findings illustrate that high doses of ethanol could damage the skin barrier. Thus, for the in vivo study, 12% of ethanol was selected which is in the range of the typical addition in dermal creams and which do not interfere with the formulation when exchanged by water. The formulations were applied by a dispenser guaranteeing a sterile product even without ethanol.

### Pilot study – in vivo investigation

**Fig. 5:**
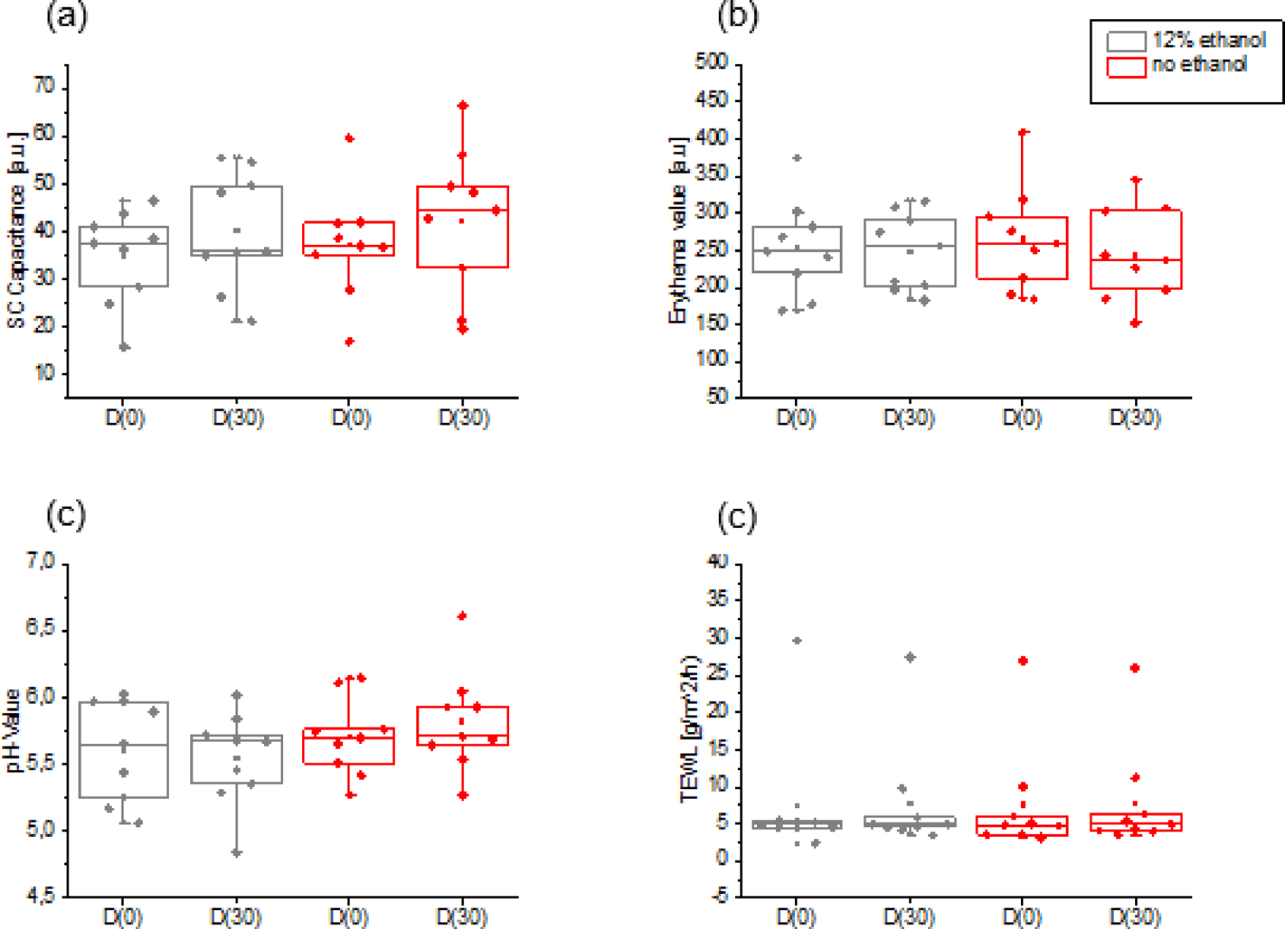
(**a**) Skin Capacitance/SC hydration, (**b**) skin erythema, (**c**) pH-value, (**d**) transepidermal waterloss and their absolute change within 30 days of twice daily cream application of ethanol-free and 12% ethanol containing creams. The gray graphs represent the ethanol-containing formulation and the red graphs the ethanol-free formulation. Neither for the individual creams nor in comparison with each other is there any difference between ethanol-containing and ethanol-free formulations in their effect on the measured parameters *n* = 9; Boxplot with the median (horizontal line within the box) and the mean (non-filled square). Upper and lower whiskers indicate the minimum and maximum within 1.5 times the interquartile range.

**Fig. 6:**
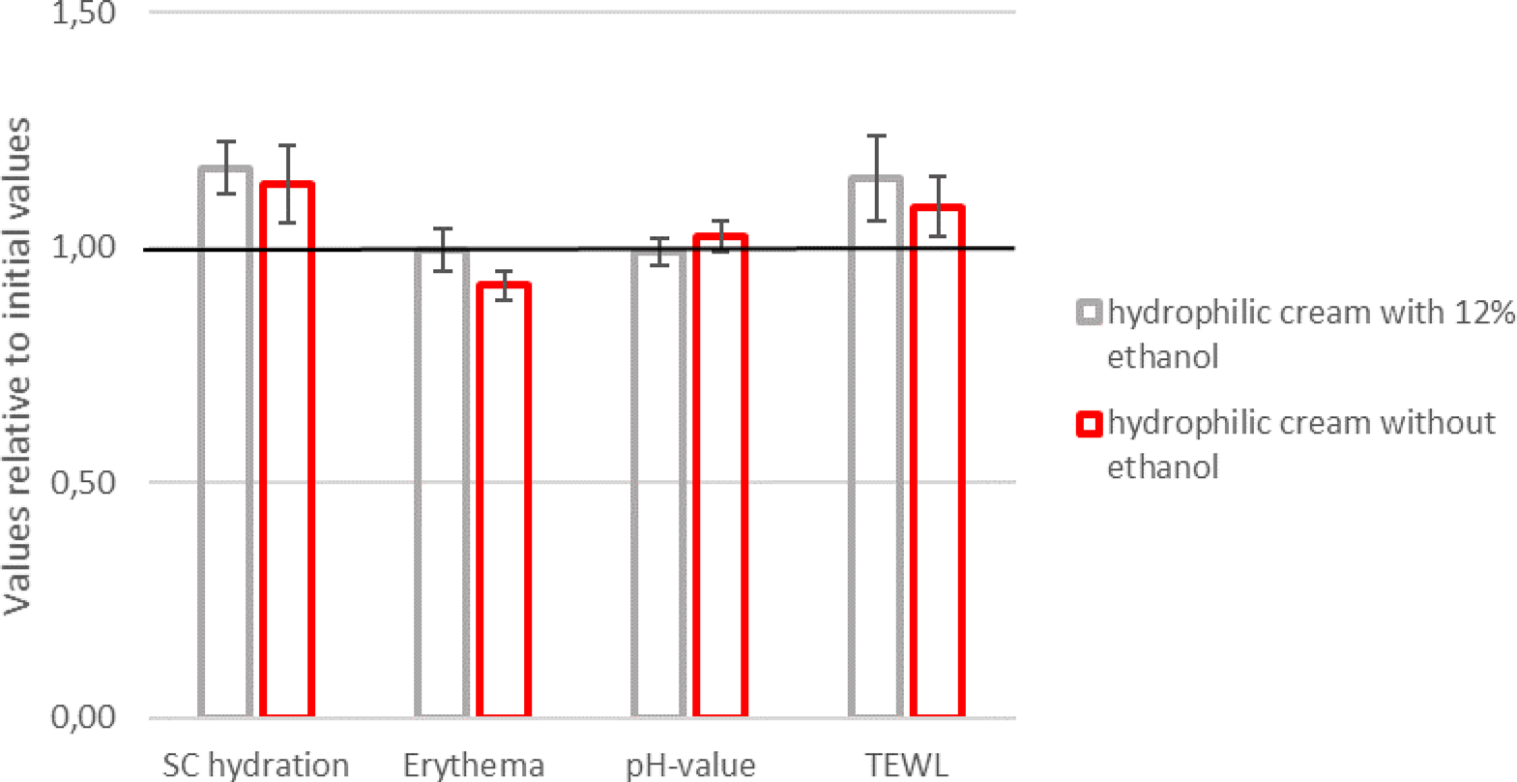
Comparison of the values relative to initial values after 30 days application of ethanol-free and 12%-ethanol containing cream. In relative comparison, both creams show a non-significant increase in SC hydration with constant erythema and pH value. The TEWL appears to be relatively higher with the ethanol-containing cream than with the ethanol-free base cream (mean ± SEM), *n* = 9.

After thirty days of application of the ethanol-containing cream, there was no significant difference concerning the change in SC hydration compared to vehicle. Both creams increased the SC content equally (5,6). Similarly, on non-inflamed atopic skin (i.e. non-lesional), an increase in the erythema value was not detected (Figs. 5 and 6). There was also no change in the local SCORAD score. The local SCORAD score for the antecubital fossae was 0 for eight patients without acute eczema at the beginning and the end of the study and thus showed clear skin findings. The patient with bilateral eczema suffered an acute deterioration in the overall condition of the skin and had to terminate the study early due to a generalized exacerbation of the AD. The pH value of the skin remained constant for 30 days in all patients after application of the cream with ethanol and the vehicle (Figs. 5 and 6). Finally, the measurements of transepidermal water loss before and after application of the creams also showed no significant difference (Figs. 5 and 6).

The ethanol containing cream seems to increase the TEWL relatively more than the ethanol-free formulation (Fig. 6). This is due to a statistical outlier, which is not removed and increased from a TEWL of 6,17 g/m^2^/h (± 1.24, SD) to 10,88 g/m^2^/h (± 1,58, SD). This can be considered a clinically plausible TEWL for the antecubital fossa, even if a relative increase of 76% is observed. Removing this outlier would show that the ethanol-containing cream would then cause an average increase of 10% (± 2,7, SD) compared to the ethanol-free cream with 6% (± 2.1, SD) (data not shown).

None of the initially non-inflamed skin areas of the patients experienced an exacerbation during the study period and the dietary and stress habits of all volunteers remained constant.

One patient with initial mild eczema in the antecubital fossa (SCORAD score of 23.8) experienced a general exacerbation of eczema during the study period. The eczema worsened to a SCORAD score of 71, which corresponds to severe atopic eczema. At the same time, new spots appeared on the face and neck. This led to an early termination of the study after 24 days. Both elbows were equally affected. Due to the general exacerbation, a general trigger can be assumed since the patient had different food allergies; an influence of the one-sided 12% ethanol application on the exacerbation could not be determined.

**Fig. 7:**
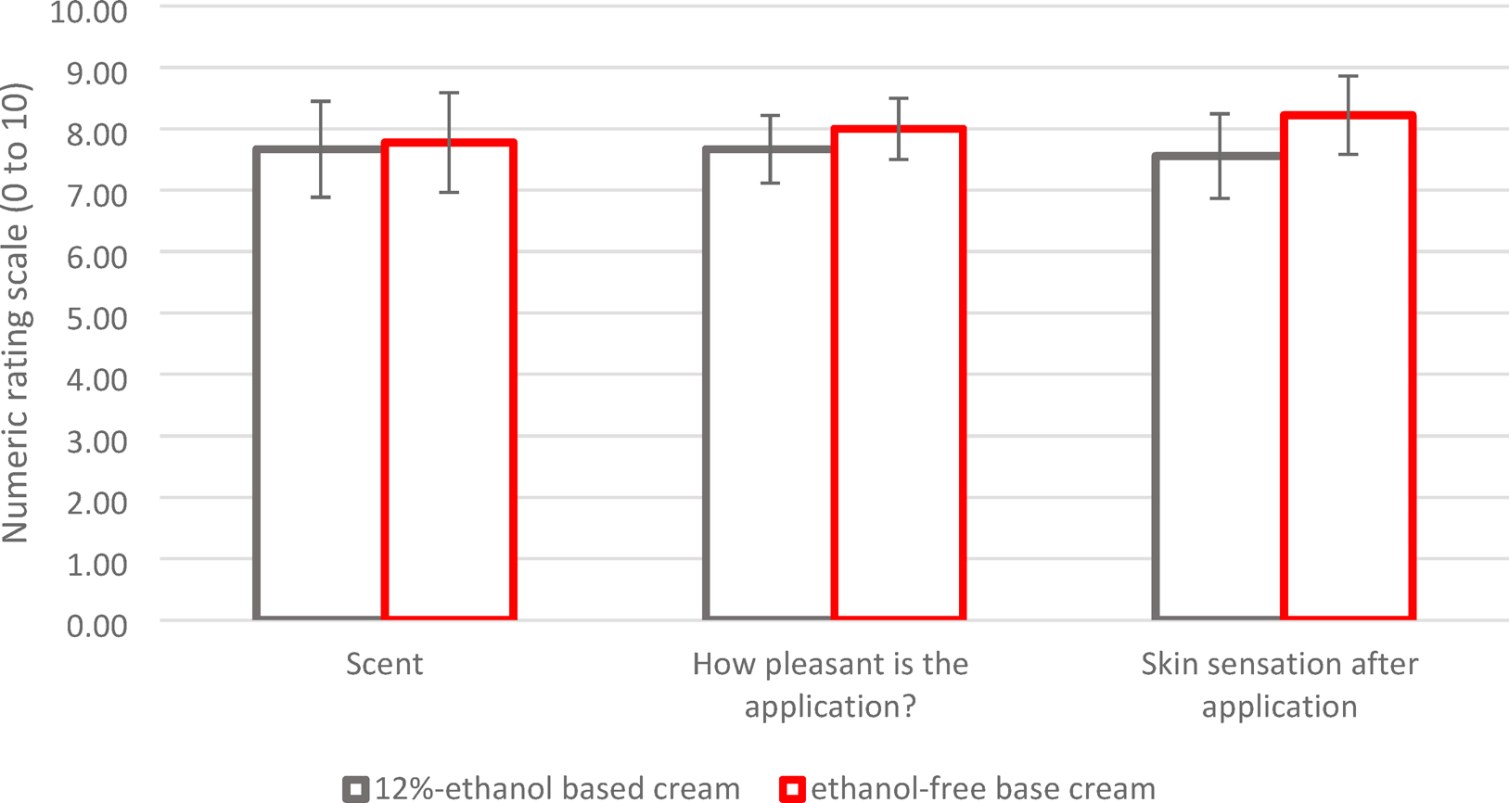
Sensory and subjective perception. There are no noticeable differences (0 - negative, 10 – positive), in scent, applicability and skin comfort after application of the ethanol-containing formulation compared to the ethanol-free formulation (mean ± SEM, *n* = 9).

There was no noticeable difference between the two creams, only the smell of the ethanol-containing cream was described as alcoholic by some patients, the smell disappeared within a few minutes of application. In the subjective perception of the two creams, there were no significant differences (Fig. 7).

Compliance with twice-daily application (morning and evening) averaged 95.74% (SD ± 2.5%).

### Microbiome analysis

**Fig. 8:**
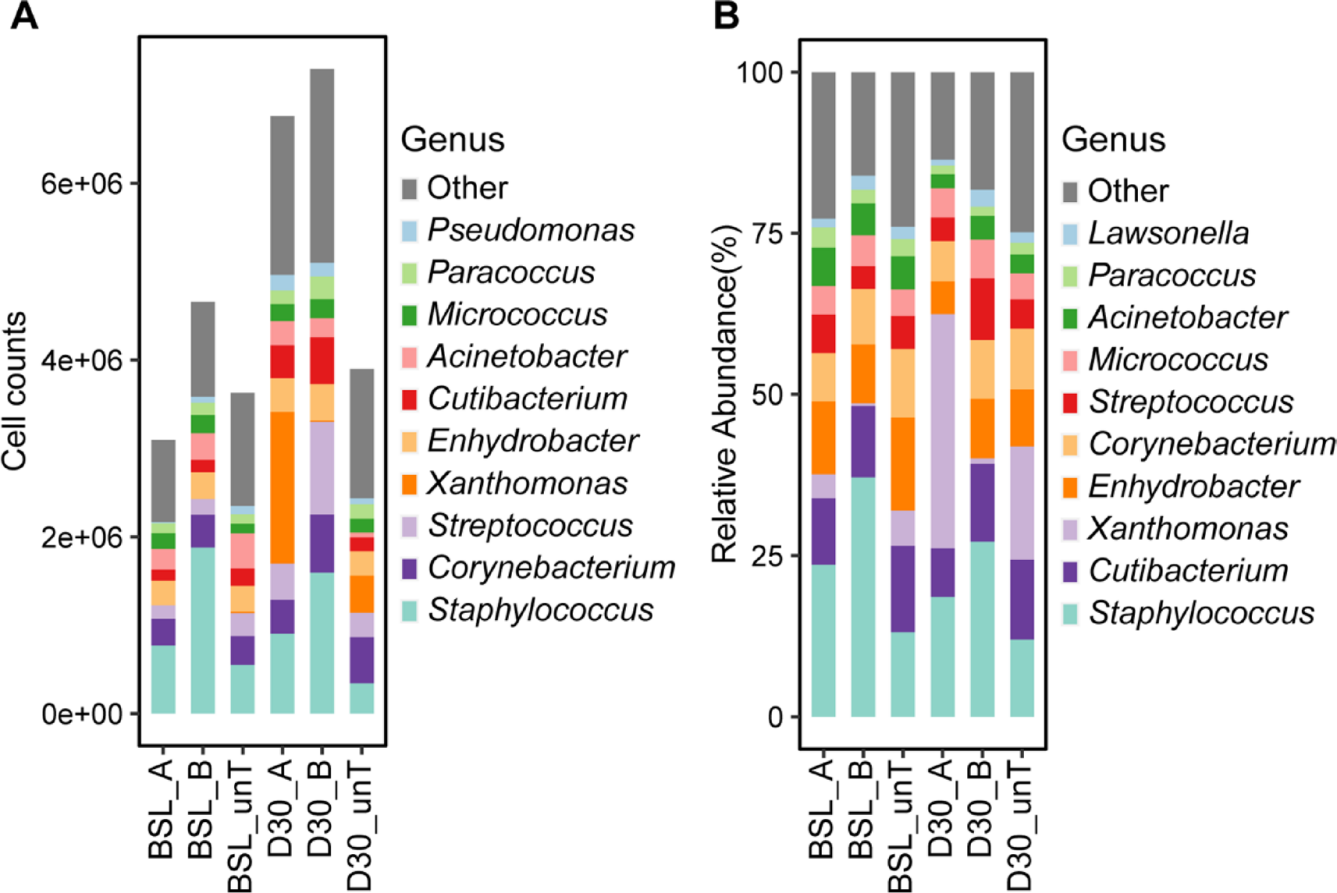
The mean quantitative estimates and relative abundance of most abundant genera (top 10). (**A**) The quantitative estimates of the 10 major genera in the skin microbiota; (**B**) The relative abundance of the 10 major skin microbes at the genus. Different colors indicate different genera.* BSL*  Baseline,* D30*   after 30 days,* unT*  untreated; A contains 12% ethanol, B is ethanol-free.

We first evaluated the bacterial load based on the QMP, and no significant difference was observed across all groups (Fig S2). The mean quantitative estimates for individual bacteria showed that the genera Staphylococcus, *Corynebacterium* and *Streptococcus* are the skin predominant microbes at the treated sites in the antecubital fossae, and also the control site (the biceps region of the upper arm) (Fig. 8A, Fig S3A). Notably, the genus Xanthomonas appears to be abundant only after 30 days of application of the ethanol-containing cream (Fig. 8A, Fig S3A). The similar patterns were observed in the analysis of relative abundance data (Fig. 8B, Fig S3B).

**Fig. 9:**
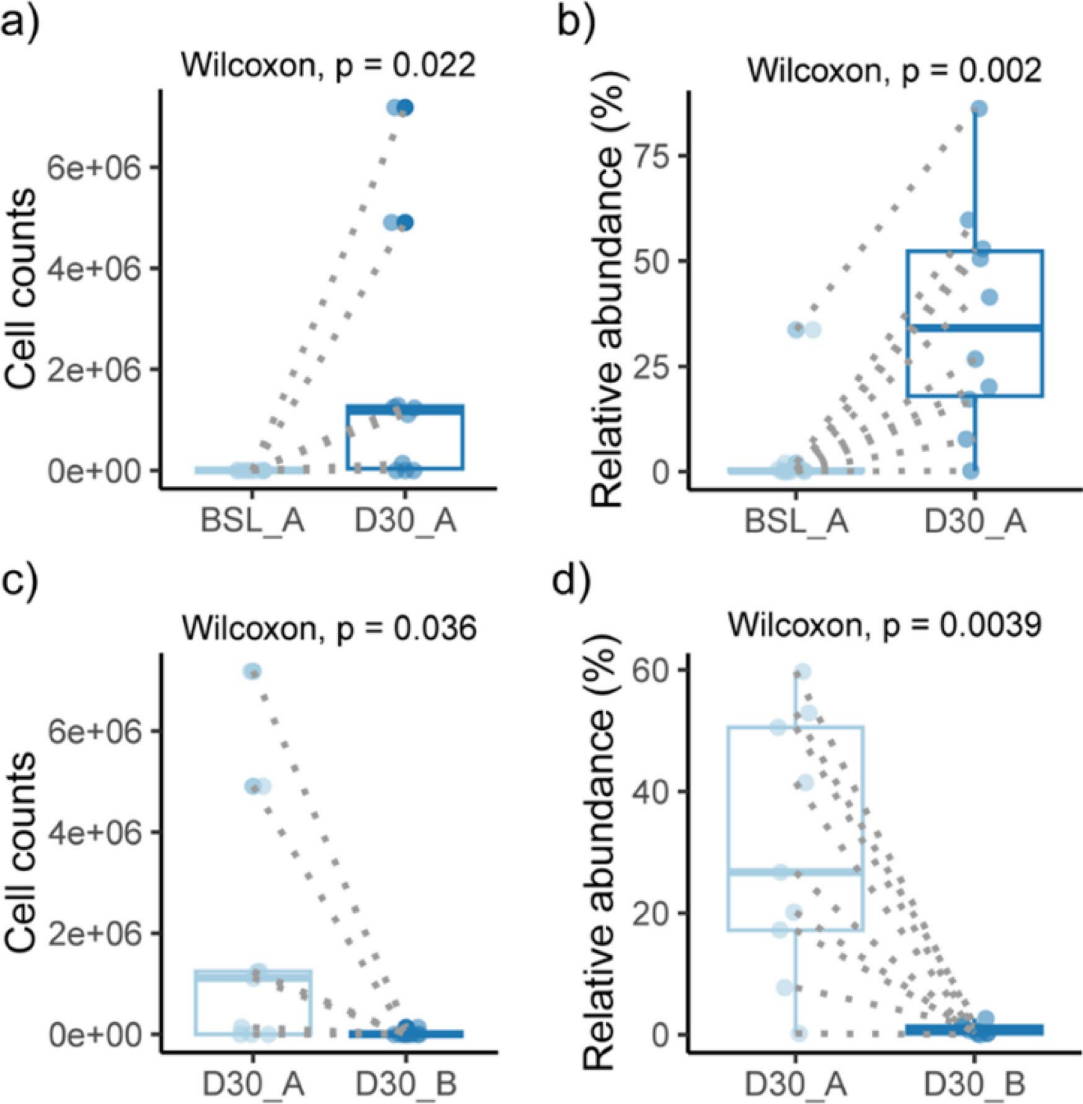
The quantitative estimates from QMP and original relative abundance of genus Xanthomonas. (**a**,** b**) Absolute cell count and relative abundance before and after application of ethanol-containing cream. (**c**,** d**) Comparison of the absolute cell count and relative abundance after 30 days of application of the ethanol-containing and ethanol-free cream. After 30 days of application of an ethanol-containing cream, the abundance of Xanthomonas is significantly higher compared to baseline and an ethanol-free cream.* BSL*  Baseline,*D30 * After 30 days; A contains 12% ethanol, B is ethanol-free.

Overall, the analysis of the local skin microbiota revealed that in almost all individuals, the richness (number of different ASVs) at the treated sites in the antecubital fossae was lower than at the control site (Fig S4), although the difference is not significant. No difference was seen between the left and right side of the antecubital region based on both alpha and beta diversities as well (Fig S5). The skin microbiota was also checked before and 30 days after two cream application, and the diversity indices show no significant difference for both ethanol-containing and ethanol-free creams, together with no difference between the two groups treated with these two creams after 30 days (Fig S6,S7). However, the quantitative estimates from QMP and relative abundance of the genus *Xanthomonas* both show a significantly higher level in the skin microbiota with the treatment of cream A, which refers to the ethanol-containing cream (Fig. 9).

## Discussion

The ex vivo evaluations of the present study showed slightly negative influence of ethanol on the healthy skin barrier with partial dehydration and barrier loss. These effects increased proportionally to the ethanol concentration. This agrees with the findings of Gurtovenko et al.^8^. An ethanol concentration of 25% can change the structure of lipids in the skin while concentrations above 58% cause the formation of pores in lipid layers that enable easier penetration of active ingredients.

The lipid washouts identified here represent only one of several mechanisms contributing to the increased penetration and autofluorescence seems particularly effective for visualizing this process^9^. However, confirmation with complementary techniques such as electron microscopy or lipidomics are necessary to validate the lipid-washout explanation and to achieve a more comprehensive understanding^35–39^. Further analysis is especially necessary to elucidate how ethanol interferes with the lamellar bilayers of the stratum corneum and to arrive at a definitive assessment.

Ethanol can penetrate the tissue which illustrate the changes in autofluorescence. These autofluorescence findings should be validated in subsequent studies to strengthen the conclusions presented here. The data are consistent with findings obtained using microdialysis. This is shown by the penetration of ethanol into the skin in an infinite dose experiment^40^.

The ex vivo investigations of the present study show that non-lesional skin could be negatively influenced when ethanol in concentrations higher than 15% is added to skin care products. One very important distinction must be made with regard to the carrier substance used. While ethanol in aqueous solution showed these effects, the effects observed here may not occur in cream formulations due to compensation by means of skin barrier-strengthening and moisturizing additives.

Thus, to evaluate this in vivo, a pilot study was performed containing 12% of ethanol compared to a similar formulation where ethanol was substituted by water.

Even though high concentrations of ethanol/water mixtures above 15% seem to reduce SC thickness (SCT), the application of 12% ethanol in a moisturizing cream seems to have no negative influence on non-lesional skin of atopic patients.

This result underlines the importance of the galenic formulation: an aqueous solution interacts differently with the skin than a cream. Although our study did not aim to directly compare the two vehicles, it shows that the results obtained with an aqueous solution cannot simply be transferred to cream formulations and need to be confirmed in further studies.

The initial mean values of the skin parameters are typical for non-lesional AD skin. The pH and erythema values are slightly higher and the skin hydration is lower than in non-AD population^41–44^. The TEWL values measured in this study do not appear to be substantially elevated compared to the expected values for healthy skin. However, a direct comparison is not possible, as no control measurements were conducted. Consequently, physiological TEWL values under the specific conditions of this study were not determined^45–48^.

One possible explanation for the low TEWL values could be the study population. It was mainly AD patients without acute eczema who were included. Relapse frequency per year was not recorded; only atopic diathesis and a history of eczema in the past were recorded. This could have led to the inclusion of patients with less severe symptoms. Another plausible reason for the almost healthy TEWL values could have been the inclusion of intrinsic endotypes. These do not show increased TEWL values despite clinically similar eczema as in extrinsic endotypes^49,50^. The general distribution of extrinsic to intrinsic AD assumes a ratio of 4:1, so that one in five patients belongs to the intrinsic endotype^49,51^. In a study population of 9 patients, this ratio may be skewed in favor of the intrinsic types, especially in subjects without eczema.

The present study was able to show in a small group of AD patients during an inflammation-free interval that frequently used concentrations of ethanol (10–15%) in cosmetic creams do not pose any relevant risks for users. In this case, the investigated concentration of 12% did not show any adverse effects on the skin physiology of the patients. The present study used a concentration of 12%, at which no penetration-enhancing effects but the preservative properties can be expected^7^. This means that we are at the threshold of enhanced drug delivery in terms of concentration. Another important aspect regarding the safe use of ethanol in skin care creams is its frequent use in products^6^. In particular, in the case of sunscreens, which often rely on ethanol, it should be assumed that AD patients come into contact with ethanol in skin care products and apparently without any major incidences of eczema flare-ups that would be described in the literature. The concentrations used are often higher than 12% ^52–54^.

In general, the study showed that ethanol in a skin care cream does not reduce stratum corneum (SC) hydration. Even if the ex vivo study assume a drying effect, it is important to point out that the ex vivo studies were carried out with ethanol/water solutions and the in vivo study with a cream formulation. This indicates that a drying effect of ethanol seems to be neutralized by the formulation in a moisturizing cream containing glycerol and different oils which are known to improve the skin hydrolipidic balance^55–57^. There was an insignificant increase in the absolute values for skin moisture under the influence of the ethanol-containing cream. In addition, it appears to have no significant effect on the erythema value or the pH value. The TEWL also remains constant in non-inflamed skin of atopic patients after a treatment with a 12%-ethanol containing cream. A difference between the ethanol-free and ethanol-containing base was not detectable in the study conducted. A slight barrier-damaging or drying effect caused by ethanol solutions, as observed in the ex vivo studies, may not be observed in skin care creams due to the use of hydrating and barrier-strengthening agents to compensate^58–60^. The hydrophilic base cream used in this study differs from more occlusive formulations often recommended to patients with atopic dermatitis, especially in cases of chronic eczema^61,62^. This choice was primarily due to technical requirements of the airless pump dispenser and to ensure that any potential side effects of ethanol were not concealed by the protective effects of an occlusive, barrier-strengthening cream.

The patient with bilateral eczema and severe deterioration cannot be included in the results of this study. The pathophysiology of the atopic dermatitis is strongly linked to triggers, so that the general worsening of the skin condition is more likely to be associated with a generalized flare-up than with participation in the study. After the study, the patient underwent dermatological treatment and was dependent on several weeks of topical glucocorticoid therapy. Interestingly, there was no difference in the treated areas in this patient, so it can be assumed that ethanol in the emollient has no relevant influence on the skin physiology and the symptoms of AD. This effect also needs to be confirmed in further research.

Of the eight patients with AD who were free of inflammation, none showed any adverse effects. In contrast, the patient with active eczema experienced worsening of her symptoms. However, ethanol as a preservative is an unlikely cause, as the worsening was bilateral and generalized. A reaction to the cream formulation itself appears more plausible. In particular, an intolerance to the emollients in the base cream could explain the bilateral worsening. Furthermore, the presence of new eczema on other areas of the skin corroborates the suspicion of generally triggered exacerbation rather than adverse reaction to the therapy^63^.

Excluding the patient with active exacerbation as a statistical and clinical outlier, there are also no significant differences between the two cream formulations. Both, after simple statistical tests and after multiple testing correction, there were no significant changes in the skin physiological parameters.

AD patients, as one of the most vulnerable consumer groups for skin care products, appear to tolerate ethanol in skin care creams. At the same time, sensitive skin syndrome is playing an increasingly important role and also affects non-AD patients^64^. By that, a safe applicability regarding the skin microbiome and skin physiology could be deduced for larger skin-healthy groups that tend to skin sensitivity.

In addition to alcohols, parabens play an important role in the preservation of cosmetics^65^. It is important to note that parabens do not have the highest reputation and the concern of hormonal interactions caused by long-term absorption through the skin still exists. Nevertheless, parabens are very likely safe in cosmetics and are a cheap preservative. Still, they can also induce allergies^65–68^. Nevertheless, the present study shows that ethyl alcohol can be a safe substitute for parabens on atopic skin as long as the possible hazard of parabens is not finally resolved.

Other common preservatives and representants of the group of alcoholic preservatives such as benzyl alcohol and phenoxyethanol are better studied than ethanol. Phenoxyethanol is one of the best-studied preservatives in cosmetics and can be classified as safe^66–70^. However, this also applies above all to normal skin. Studies on explicitly atopic skin are also lacking for phenoxyethanol. The present study therefore attempts to offer a safe preservative, namely ethanol, in cosmetics for this group of consumers.

Although benzyl alcohol is a potential contact allergen and sensitizer, its use is safe in cosmetics for the large group of skin-healthy people^71,72^. However, this does not necessarily apply to atopic skin, which is more susceptible to contact allergies due to the impaired skin barrier. this is the reason why benzyl alcohol should be avoided by many patients with atopic diathesis. Contact-allergenic preservatives such as methylchloroisothiazolinone (MCI) and methylisothiazolinone (MI) should be avoided in favor of phenoxyethanol or ethanol, for example^73–76^.

Some alcohol-based preservatives were found to have a positive effect on existing skin dysbiosis^66^. However, much more research needs to be done in this area so that microbiome research of the skin can make further progress. The question of whether preservatives can also be useful in the supportive treatment of atopic dermatitis is an interesting new research question. It remains an important topic to counter multifactorial atopic dermatitis with several pathways of therapy^77^. In addition to the currently widespread anti-inflammatory and barrier-strengthening therapy strategies, a microbiome-stabilizing strategy could be the breakthrough for even longer inflammation-free intervals and more effective topical therapies that can be carried out easily, regularly and inexpensively by affected patients. Work is ongoing on new antimicrobial agents that should lead to a reduction in the frequency and intensity of flare-ups^78^.

The alpha diversity of the non-lesional AD skin found here is consistent with the findings in the literature^14,79,80^. There is a dominance in absolute abundance of *staphylococci* compared to people with healthy skin, which, as a control, was not recorded here. The increased occurrence of *Streptococcus species (spp.)* has also been described on non-inflamed skin of AD patients^81,82^.

From a microbiological point of view, the application of 12% ethanol in an emollient to the skin does not generally appear to cause any major shifts in the microbiome. A more precise comparison of the microbiome shift would be more accurate with a control point in another moist or sebaceous zone such as the forehead.

It is known that phenoxyethanol, as a classic representative of alcoholic preservatives, has the least influence on the resident flora compared to other preservatives^83^. The present study suggests that ethanol, at a concentration of 12%, shows similar to phenoxyethanol no relevant changes of the skin microbiome of AD patients.

However, it was observed that *Xanthomonas spp*. increased significantly with the use of an ethanol-containing cream at the investigated concentration. Amar et al. previously showed this observation in a murine AD model; nevertheless, they did not explore the significance of the increased Xanthomonas abundance^84^. Xanthomonas spp. are mainly known as plant-pathogenic bacteria but Xanthomonas DNA has also detected on human skin^82,85–88^. Increased level of bacteria of the genus *Xanthomonas* were found on the skin of psoriasis patients after balneotherapy^85^. Further studies have to evaluate whether an increase in *Xanthomonas* may be associated with a therapy success of psoriasis or even AD. An expansion of the cutaneous microbiome appears achievable; given the inherently low microbial diversity characteristic of atopic dermatitis, such diversification is likely to be beneficial. Previous small-scale studies have consistently viewed an increase in *Xanthomonas spp*., usually accompanied by reduced staphylococcal colonization, as a favorable shift and have therefore regarded these bacteria as beneficial commensals in atopic dermatitis. Our data, however, are insufficient to confirm or refute this conclusion^89–91^.

Nonetheless, it should be considered that Xanthomonas species may have been introduced through the cream formulation. It is known that Xanthan gum, which is used as an emulsifier in the cream formulation, is produced by Xanthomonas campestris strains^92^. Murphy et al. stated that in their study the detection of DNA from *Xanthomonas spp.* was mainly due to contamination caused by residual *Xanthomonas* DNA from the xanthan gum^93^. However, in the present pilot study, a significant increase in *Xanthomonas spp*. was only found on the ethanol-containing cream side, not after use of the ethanol-free formulation. Moreover, *Xanthomonas spp.* was also detected on the untreated skin indicating that the detected *Xanthomonas* DNA is not due to residual DNA from the cream. This was further confirmed by the absence of significant microbial DNA amounts in the cream as analyzed by PCR analysis. Furthermore, our microbial DNA analysis removes at first free contaminating DNA and subsequently isolates DNA only from intact bacteria. Thus, we believe that the detection of *Xanthomonas spp.* in our study is not based on residual contaminating DNA from the cream formulation.

In conclusion, ethanol at a concentration of 12% was found to be safe and well-tolerated in patients with atopic dermatitis (AD) during the non-inflammatory stage when used regularly in a hydrophilic cream. It did not significantly affect the skin barrier, including trans-epidermal water loss (TEWL), stratum corneum (SC) hydration, skin erythema, or pH levels.

However, when using ethanol-containing aqueous solutions with concentrations of more than 15%, a slight drying or barrier-weakening effect was observed in ex vivo tests. This effect was not observed in patients using a skin care cream formulated with 12% ethanol as a preservative. Additionally, only minimal changes in bacterial species were observed after regular application of the ethanol-based cream, and no negative impact on the local skin microbiome was found. A local increase in the genus *Xanthomonas* was observed after treatment with the ethanol-containing cream; clinically, no change was observed in this context. Thus, the role and relevance of *Xanthomonas spp.* remains unclear. Further research is needed to clarify the spread of this organism and its effects.

The subjective sensation of application was rated similarly to that of the vehicle cream without added ethanol, indicating no significant difference in the comfort of use.

Further research is needed to clarify and verify non-negative effects of ethanol in skin care creams.

## Supplementary Information

Below is the link to the electronic supplementary material.

Supplementary Material 1

## Data Availability

Underlying raw data and images are available from the corresponding author upon reasonable request.
