# Supplementary material for: A pilot study on the cutaneous effects of ethanol in a moisturizing cream on non-lesional skin of patients with atopic dermatitis
Source: Sci Rep. 2025 Sep 15;15:32536. doi: 10.1038/s41598-025-18487-9 (PMC12436639; doi:10.1038/s41598-025-18487-9)
Supplement: Supplementary file 1 — Supplementary Material 1 [file 41598_2025_18487_MOESM1_ESM.pdf]

Supplementary Figures

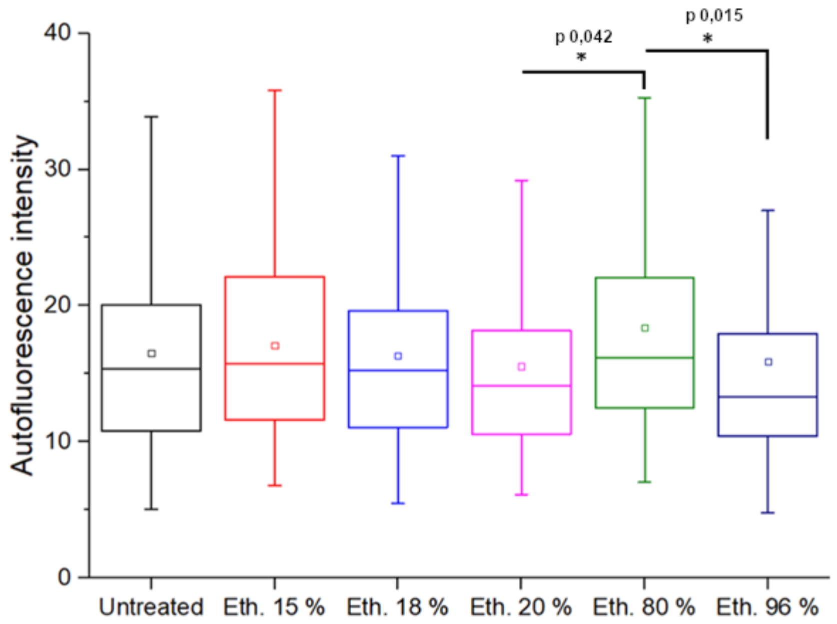

*Figure S1 Autofluorescence after ethanol application with concentrations of 15 to 96%, n=3 porcine ears with 280 different measured points per ear, * p ⩽ 0.05. After multiple correction, there is a significant increase of autofluorescence between the 20% and 80% ethanol treated skin. With increased ethanol concentration to 96% the autofluorescence is significantly lower than with 80% treated skin.*

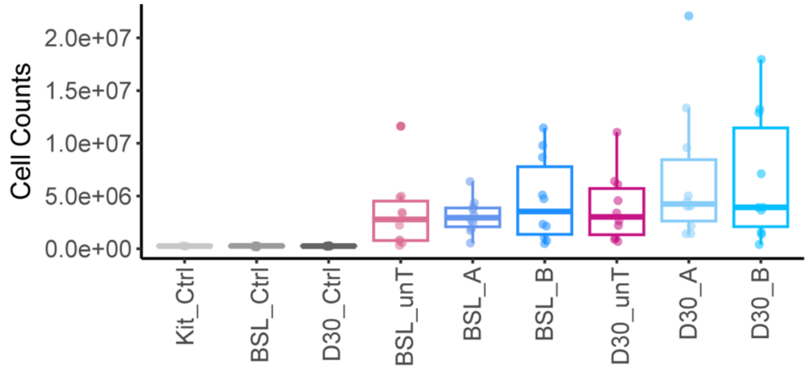

Figure S2 The quantitative estimates across all groups. Ctrl = the negative controls for DNA extraction or sampling environments; BSL = Baseline, unT = untreated; A contains 12% ethanol, B is ethanol-free.

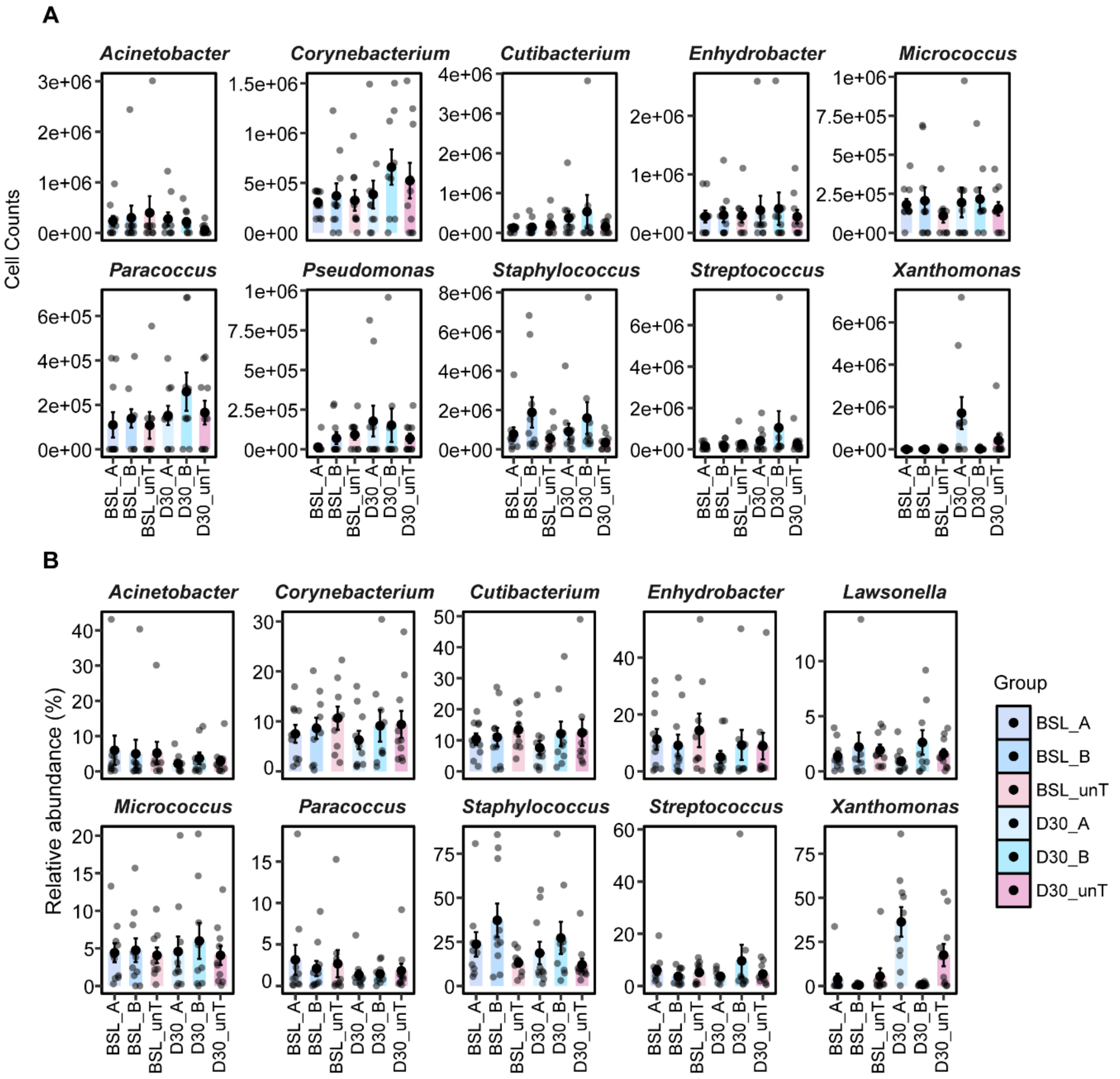

Figure S3 The mean quantitative estimates and relative abundance of the ten representative microbes at the genus. (A) The averages of quantitative estimates of the most abundant genera with error bars based on standard errors. (B) The averages of relative abundance of the top 10 abundant skin microbes at the genus level with error bars based on standard errors. Different colors indicate different groups.

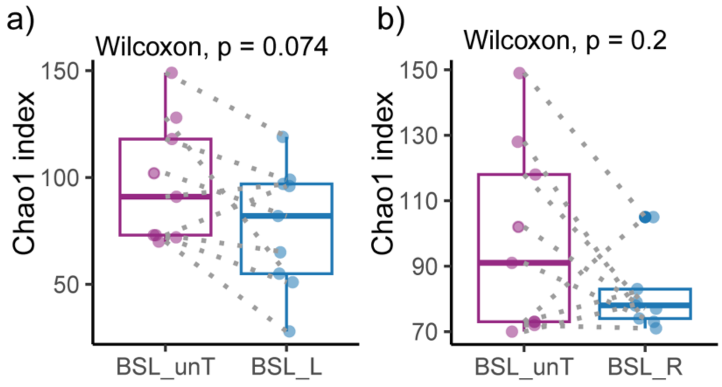

Figure S4 The richness (number of different amplicon-sequence variants, ASVs) at the treated sites in the antecubital fossae and control site in the bicep's region of the upper arm. BSL = Baseline, unT = untreated. The P values were computed by non-parametric Wilcoxon test.

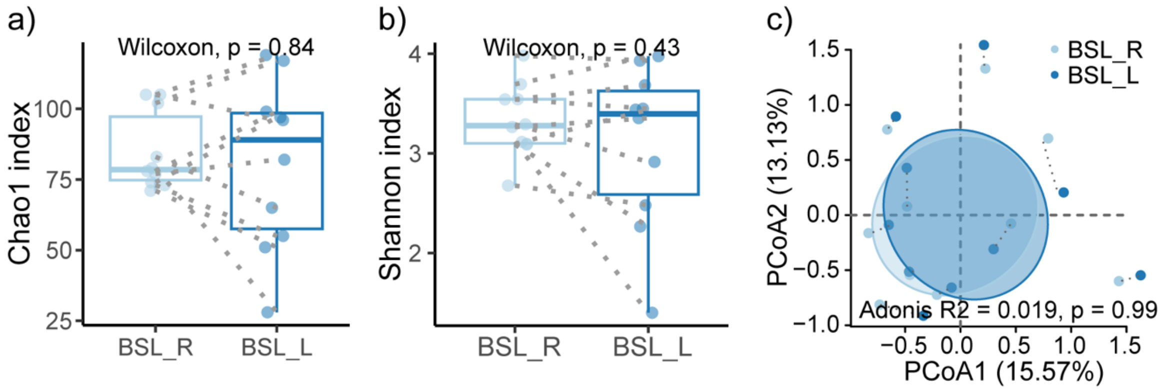

Figure S5 The alpha and beta diversities of the skin microbiota at the treated sites in the antecubital fossae. The richness assessed with Chao1 index (a). The evenness assessed with Shannon index (b). The composition of skin microbiota community assessed with Principal Coordinate Analysis (PCoA) based on Bray-curtis dissimilarity (c). The P values were computed by non-parametric Wilcoxon test and PERMANOVA.

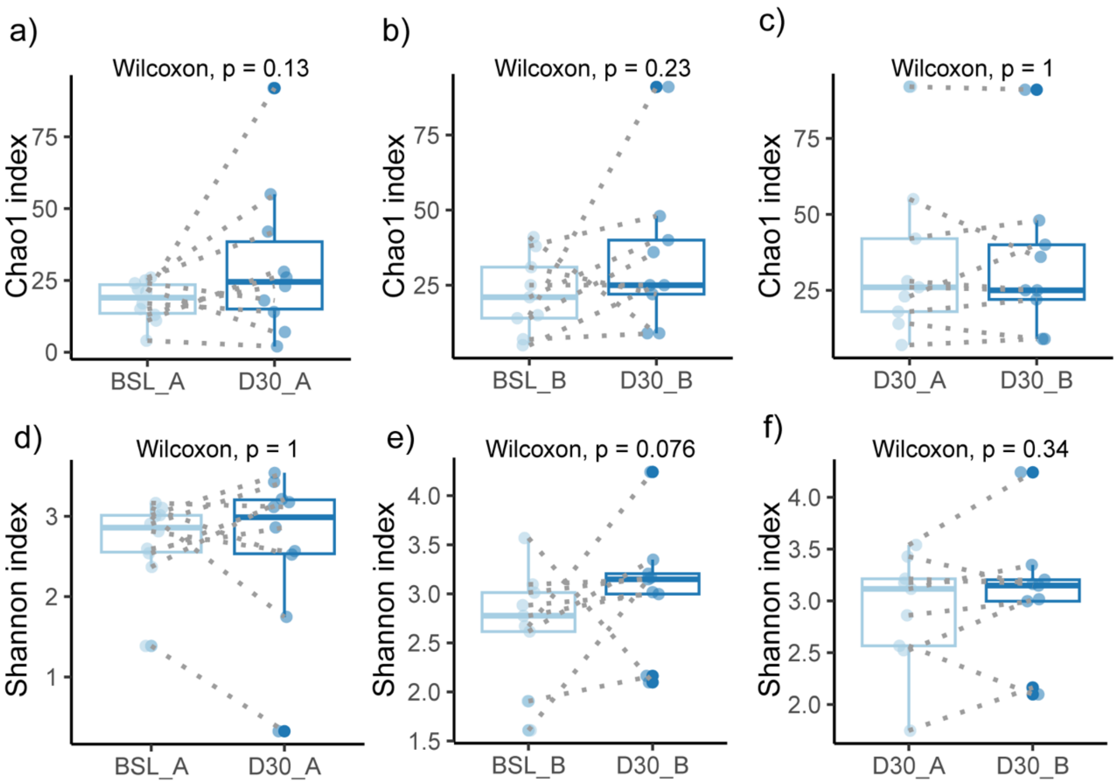

Figure S6 The alpha indices of the skin microbiota at the treated sites in the antecubital fossae before and 30 days after creams application. The richness assessed with Chao1 index (a-c). The evenness assessed with Shannon index (d-f). A contains 12% ethanol, B is ethanol-free. The P values were computed by non-parametric Wilcoxon test

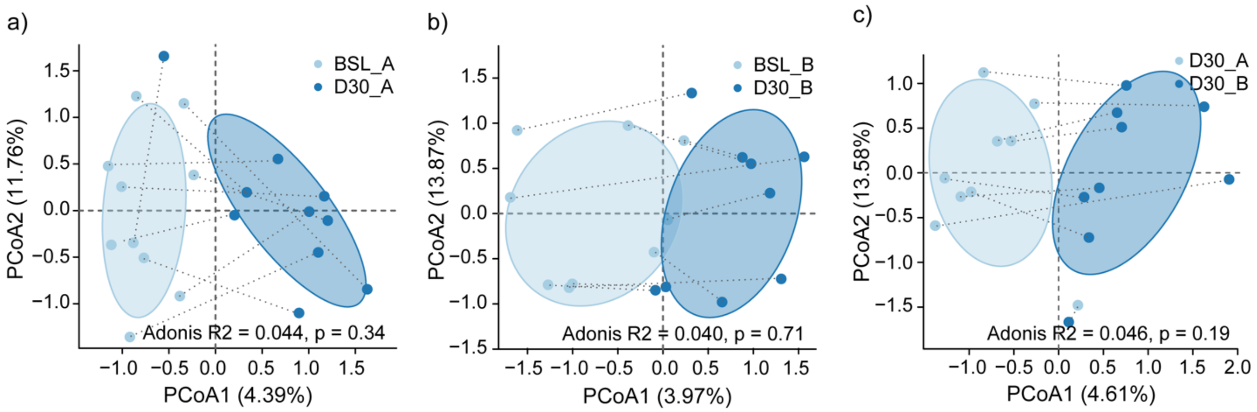

Figure S7 The PCoA of the skin microbiota at the treated sites in the antecubital fossae before and 30 days after creams application. The composition of skin microbiota community was assessed with PCoA based on Bray-curtis dissimilarity. The P values were computed by PERMANOVA. A contains 12% ethanol, B is ethanol-free.
